# Genetic analysis of teat number in pigs reveals some developmental pathways independent of vertebra number and several loci which only affect a specific side

**DOI:** 10.1186/s12711-016-0282-1

**Published:** 2017-01-04

**Authors:** Gary A. Rohrer, Dan J. Nonneman

**Affiliations:** U.S. Meat Animal Research Center, USDA, Agricultural Research Service, Clay Center, NE USA

## Abstract

**Background:**

Number of functional teats is an important trait in commercial swine production. As litter size increases, the number of teats must also increase to supply nutrition to all piglets. Therefore, a genome-wide association analysis was conducted to identify genomic regions that affect this trait in a commercial swine population. Genotypic data from the Illumina Porcine SNP60v1 BeadChip were available for 2951 animals with total teat number (TTN) records. A subset of these animals (n = 1828) had number of teats on each side recorded. From this information, the following traits were derived: number of teats on the left (LTN) and right side (RTN), maximum number of teats on a side (MAX), difference between LTN and RTN (L − R) and absolute value of L − R (DIF). Bayes C option of GENSEL (version 4.61) and 1-Mb windows were implemented. Identified regions that explained more than 1.5% of the genomic variation were tested in a larger group of animals (n = 5453) to estimate additive genetic effects.

**Results:**

Marker heritabilities were highest for TTN (0.233), intermediate for individual side counts (0.088 to 0.115) and virtually nil for difference traits (0.002 for L − R and 0.006 for DIF). Each copy of the *VRTN* mutant allele increased teat count by 0.35 (TTN), 0.16 (LTN and RTN) and 0.19 (MAX). 15, 18, 13 and 18 one-Mb windows were detected that explained more than 1.0% of the genomic variation for TTN, LTN, RTN, and MAX, respectively. These regions cumulatively accounted for over 50% of the genomic variation of LTN, RTN and MAX, but only 30% of that of TTN. *Sus scrofa* chromosome SSC10:52 Mb was associated with all four count traits, while SSC10:60 and SSC14:54 Mb were associated with three count traits. Thirty-three SNPs accounted for nearly 39% of the additive genetic variation in the validation dataset. No effect of piglet sex or percentage of males in litter was detected, but birth weight was positively correlated with TTN.

**Conclusions:**

Teat number is a heritable trait and use of genetic markers would expedite selection progress. Exploiting genetic variation associated with teat counts on each side would enhance selection focused on total teat counts. These results confirm QTL on SSC4, seven and ten and identify a novel QTL on SSC14.

**Electronic supplementary material:**

The online version of this article (doi:10.1186/s12711-016-0282-1) contains supplementary material, which is available to authorized users.

## Background

Genetic selection for increased litter size in pigs has resulted in many sows giving birth to more live piglets than they are capable of nursing. The competition for teats leads to increased pre-weaning mortality due to crushing and starvation [1]. Therefore, selection on teat number has begun to ensure that sows can nurture all of their piglets [2]. Number of piglets born in the largest 25% of litters in purebred Danish Large White and Landrace exceeded 18 in sows born in 2009 [3], which indicates that the number of piglets born was larger than the number of teats for a substantial proportion of litters. Number of teats in pigs is a variable and heritable trait. Number of teats differs between breeds, for example [4–6], and is moderately heritable [7–10]. Numerous genome scans have been conducted for number of teats in pigs (QTLdb; http://www.animalgenome.org/cgi-bin/QTLdb/SS/index), yet to date, few causative genes (or variants) have been discovered.

Most early studies used crosses between Meishan and occidental F_2_ swine populations and detected the largest QTL on either *Sus scrofa* chromosome SSC1 or 7 [6, 11, 12]. These QTL on SSC1 and seven coincided with QTL for vertebra number or carcass length, which led to the hypothesis that vertebra and teat number were controlled by common genes [6, 13]. Putative causative genetic variations for vertebra number in the *NR6A1* gene [14] on SSC1 and the *VRTN* gene [15] on SSC7 have been associated with variation in teat number in Meishan × occidental cross populations [6, 12]. *VRTN* has also been associated with teat number in commercial swine populations [13, 16, 17].

While the presence of mammary glands is a defining character of species in the class Mammalia, location and number of mammary glands across species are quite variable [18]. Mammary glands commonly exhibit bilateral symmetry [19] and variation in number of functional mammary glands within a species is relatively low. Among the farmed artiodactyl species, only pigs have thoracic/pectoral and abdominal mammary glands, in addition to the inguinal mammary glands that are present in all artiodactyls. Mice are a common model mammalian species, yet they lack abdominal mammary glands and male pups do not have any visible teats at all. A greater understanding of mammary gland development is necessary to fully exploit the genetic variation present in pigs.

In early embryonic development of mammals, three separate streaks of multilayered surface ectoderm will form a mammary line that spans from the axilla to the inguen (groin) of the embryo. Mammary line cells will either group together or regress and eventually form mammary rudiments, which can later develop into functional mammary glands. As the gland continues to form, milk canals and nipples develop, completing the process. The developmental process in mice suggests that each pair of mammary glands develops at its own pace and may be regulated by different mechanisms [19, 20]. While male mouse embryos develop mammary rudiments, these structures typically regress prior to birth [19].

Studies have shown that spontaneous events and genetic mutations can result in bilateral asymmetry of mammary development in mice. Fernández et al. [9] speculated that the observed fluctuating asymmetry in the number of nipples in pigs may be caused by disruption of co-adaptive gene complexes, which results in developmental instability. Fluctuating asymmetry has been studied in numerous species and it is often associated with increased stress or disease during critical development time periods.

Therefore, to increase our knowledge on the genetic factors that regulate mammary gland development in pigs, we conducted genome-wide association studies (GWAS) for various measurements of teat number in a composite population of commercial pigs. Individual counts of number of teats on each side were collected to evaluate bilateral symmetry and to determine if selection on total number of teats was the most effective measurement to record. Single nucleotide polymorphisms (SNPs) that were highlighted in the GWAS were then evaluated in an expanded population which contained germplasm from additional lines of commercial pigs. The results presented will be useful to enhance selection for increased lactation capacity as well as identify potential candidate genes that affect mammary gland development.

## Methods

### Data collection

#### Description of the population

The population of pigs used for this study was a ½ Landrace–¼ Duroc–¼ Yorkshire composite population that was created in 2001 and maintained as a closed population through 2010 as previously described [21]. Animals born in 2011 were from dams of this population and sired by Landrace boars from industry suppliers, while animals born in 2012 were sired by Yorkshire sires from industry sources. All pigs produced were processed at 1 day of age, when the number of teats was recorded and the tail docked and stored for DNA extraction as part of the standard operating procedure which has been approved by the USMARC IACUC committee. Animals born from May 2008 to August 2009 had number of teats recorded for left and right sides.

#### Genotypic data

Extraction of DNA from tail tissue was done using the Wizard^®^ genomic DNA purification kit for genomic DNA purification according to the manufacturer’s protocols (Promega Corp., Madison, WI, USA). Approximately 75 ng of genomic DNA was then used in the reactions for the Illumina genotyping platforms. Assays using the Illumina Porcine SNP60 BeadChip v1 were done at USMARC and the chips were scanned at USDA-ARS-BARC Bovine Functional Genomics Laboratory, while all other genotyping analyses were done at GeneSeek (Lincoln, NE, USA). In total, 2951 Landrace–Duroc–Yorkshire pigs were genotyped with the Illumina Porcine SNP60v1 BeadChip and used for the GWAS. In addition, 2502 animals from either the closed Landrace–Duroc–Yorkshire population (n = 1275) or animals sired by commercial boars (n = 1227) were genotyped using one of the other three Illumina-based genotyping platforms (Illumina Porcine SNP60v2 BeadChip; NeoGen Porcine GGP and NeoGen Porcine GGPHD) and were included in the validation phase analyses.

### Data analysis

#### Model development

The appropriate statistical model for genome-wide association was determined based on analyses that were conducted using all animals that had recorded teat counts for left and right sides (n = 6472). Evaluated phenotypes were total (TTN), left (LTN) and right (RTN) teat number. In addition, maximum number of teats on one side (MAX), left minus right side teat number (L − R) and the absolute value of L − R were also analyzed (DIF). An animal model, which fit sex and contemporary group as fixed effects, percentage of males born in the litter as a covariate and litter as a random effect, was initially run as the full model using WOMBAT [22]. Reduced models in which one effect was eliminated were run and the residual and phenotypic variances were estimated, and then these were compared with the estimated variances from the full model. The order in which effects were eliminated was based on the predicted effects and was as follows: percentage of males in the litter, sex of the animal, and lastly the random effect of litter. This procedure was conducted on all six phenotypes studied.

#### Genome-wide association analyses

The dataset for GWAS included only animals with teat count records (total or left and right side data) and that were genotyped with the Illumina Porcine SNP60v1 BeadChip (n = 2951) using GENSEL v4.61R (http://bigs.ansci.iastate.edu). However, only 1828 animals had data for individual side counts. BayesC *π* was initially run to estimate variance components and *π* for the final genome-wide association analyses for which a minimum of 4000 iterations were conducted after removing 100 iterations for burn-in. Posterior estimates of *π* were evaluated to determine if the estimate of *π* had converged. For some traits, it was necessary to run more iterations to obtain a stable estimate of *π*. Genome-wide associations were conducted running BayesC with a prior as determined in the BayesC *π* runs. A total of 41,000 iterations were performed with the first 1000 discarded for posterior summaries. A 1-Mb window approach was conducted as described by Rohrer et al. [23]. Therefore, SNPs were required to have a unique position in the current swine genome (Build 10.2; [24]) resulting in 41,148 SNPs included in the final analyses. A fixed effect for contemporary group was included for all traits. A covariate for number of copies of the B allele of SNP NV090 [15], to account for the effect of *VRTN* alleles, was fitted for TTN, LTN, RTN and MAX. Genotypes for SNP NV090 were predicted as previously described [16]. SNP NV090 was selected because it is located 6 kb upstream of the transcriptional start site for *VRTN* (based on GenBank Accession AB554652), gave very reliable genotypes in our lab and was found to be in complete linkage disequilibrium with the 291-bp insertion into the intron of *VRTN* (NV123) [23], which may actually be the causative mutation. Windows that explained more than 1% of the genomic variation are presented.

#### SNP validation

The SNP that explained the highest proportion of genomic variation for each 1-Mb window explaining more than 1.5% of the genomic variation for any count trait (TTN, LTN, RTN or MAX) was identified (see Additional file 1: Table S1) and used for this evaluation. All animals from the USMARC herd with SNP genotypes that were obtained from any of the four Illumina-based platforms were included (n = 5453). This included an additional 1275 animals from the closed Landrace–Duroc–Yorkshire population and 1227 animals sired by commercial boars. For SNPs that were not genotyped on an animal, genotypes were imputed using FImpute [25] based on information on flanking SNPs and at least three generations of pedigree data. All SNPs were fitted simultaneously by including a covariate for number of copies of the B allele. When two SNPs had a linkage disequilibrium coefficient exceeding 0.8, one of the SNPs was eliminated from the model. The only phenotypic data available for all animals was TTN. An animal model was fit using at least three generations of pedigree data and including fixed effects for contemporary group and breed of sire as well as a covariate for the effect of *VRTN* alleles (also imputed for this dataset using FImpute). Additive genetic variance was estimated in this dataset both with and without fitted SNPs to determine the percentage of genetic variation represented by the effects of SNPs. Candidate genes were selected by manually inspecting genes that were positioned within QTL regions based on the UCSC Genome Browser Gateway (www.genome.ucsc.edu/cgi-bin/hgGateway) using Sscrofa 10.2 genome build.

## Results

Descriptive statistics and estimates of heritabilities obtained from the analysis of 6472 animals with data recorded for TTN, LTN, RTN, MAX, L − R and DIF are in Table 1. The fixed effect of sex as well as the regression coefficient for percentage of males in a litter did not affect estimates of residual or phenotypic variance for any trait analyzed and were removed from the model. The random effect of litter accounted for only ~2.5% of the phenotypic variation for TTN, LTN, RTN and MAX and heritabilities of 0.41, 0.32, .023 and 0.29 were estimated, respectively. When the random effect of litter was removed from the model for the four count traits, phenotypic variance increased by approximately 3% while the estimate of additive genetic variation increased by an average of 21% and residual variance decreased by an average of 6%. Therefore, it was concluded that the random effect of litter was absorbing some of the additive genetic variation and removing this term seemed appropriate. When the random effect of litter was removed, estimated heritabilities were highest for TTN (0.49), intermediate for MAX (0.40), LTN (0.38) and RTN (0.30) and nil for L − R and DIF.

**Table 1:** Descriptive statistics and estimates of variance components for the population of animals (n = 6472) used for statistical model development

Trait	Residual variance	Additive genetic variance	Phenotypic variance	Heritability (SE)	Mean	Range
TTN	0.592	0.578	1.171	0.494 (0.038)	14.73	8 to 21
LTN	0.242	0.149	0.390	0.381 (0.037)	7.32	5 to 13
RTN	0.319	0.137	0.456	0.301 (0.035)	7.41	2 to 12
MAX	0.234	0.154	0.389	0.397 (0.037)	7.59	6 to 13
L − R	0.525	0.000	0.525	0.000 (0.003)	−0.08	−4 to 7
DIF	0.324	0.002	0.326	0.006 (0.005)	0.45	0 to 7

*LTN* left side teat number, *MAX* maximum teat number of a side, *RTN* right side teat number, *TTN* total teat number, *L* − *R* difference between LTN and RTN, and *DIF* absolute value of L − R

### Genome-wide association study

Summary statistics from the GENSEL analyses are in Table 2. Genotypic data from the Illumina Porcine SNP60v1 BeadChip [26] were available for 2951 animals with TTN records, of which a subset (n = 1828) had number of teats recorded on each side. Genotypic data for SNPs that had a unique location on the *S. scrofa* build 10.2 [24], a minor allele frequency higher than 0.05 and a call rate higher than 80% were considered, which resulted in 41,148 SNPs after editing. SNP heritabilities (i.e. genomic heritabilities) were highest for TTN (0.233), intermediate for individual side counts (0.088 to 0.115) and virtually nil for difference traits (0.002 for L − R and 0.006 for DIF).

**Table 2:** Summary statistics from GENSEL genome-wide association analysis of animals genotyped with the Illumina Porcine SNP60v1 BeadChip (n = 2951)

Trait	Genomic variance (GV)	Phenotypic variance	Genomic heritability	Number of 1-Mb windows >1% GV	Percent of GVexplained by 1-Mb windows^a^	Mean	Range
TTN	0.221	0.948	0.233	15	30.6	15.8	8 to 20
LTN	0.034	0.356	0.096	18	57.4	7.6	5 to 10
RTN	0.035	0.396	0.088	13	50.6	7.7	2 to 10
MAX	0.040	0.345	0.115	18	54.2	7.9	6 to 10
L − R	0.001	0.528	0.002	4	9.2	−0.05	−3 to 4
DIF	0.001	0.312	0.006	6	26.0	0.47	0 to 4

*LTN* left side teat number, *MAX* maximum teat number of a side, *RTN* right side teat number, *TTN* total teat number, *L* − *R* difference between LTN and RTN, and *DIF* absolute value of L − R

^a^Percentage of phenotypic variation explained by markers, as predicted by GENSEL, that was explained by SNPs contained in 1-Mb windows that exceeded the 1% genomic variation threshold

**Fig. 1:**
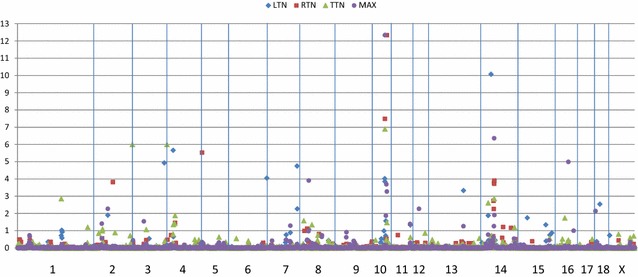
Manhattan plot of GENSEL genome-wide association analysis for count traits. *Horizontal* axis is the position on the swine genome (Build 10.2) and the *vertical* axis is the percent of genomic variation associated with each 1-Mb window

The number of 1-Mb windows that explained more than 1% of the genomic variation detected was 15 for TTN, 18 for LTN, 13 for RTN and 18 for MAX (Fig. 1). These regions cumulatively accounted for over 50% of the genomic variation in LTN, RTN and MAX, while they only explained 30% of the genomic variation in TTN. Ten 1-Mb windows were associated with more than one trait. Most notable was the chromosome SSC10:52 Mb which was associated with all four count traits, while SSC10:60 and SSC14:54 Mb were associated with three of the four count traits. The 1-Mb windows that explained more than 1% of the genomic variation are in Fig. 1. The estimated additive effects of *VRTN* were equal to 0.35 for TTN, 0.16 for LTN, 0.19 for RTN and 0.17 for MAX. Information on all 1-Mb windows that explained more than 0.40% of the genomic variation and the SNPs that are associated with the most variation are in Additional file 1: Table S1.

Several 1-Mb windows that explained more than 1% of the genomic variation for L − R (n = 4) and DIF (n = 6) were detected; however, these results are likely meaningless since the estimated genomic variation was nearly zero (Table 2). A region with a large effect on DIF was found on SSC8 between 95 and 96 Mb and accounted for over 20% of the genomic variation. Cumulatively, the regions that explained more than 1% of the genomic variation for these traits accounted for <0.2% of the observed phenotypic variation.

Thirty-six SNPs (including the SNP in *VRTN*) that were located in 32 unique 1-Mb windows were selected for the validation phase. Three SNPs were removed because they created multi-colinearity among the regression coefficients due to high linkage disequilibrium. All three SNPs were within a 1-Mb window represented by multiple SNPs, which were associated with different phenotypes. In total, the SNPs accounted for ~39% of the additive genetic variation estimated for this dataset. The effect of *VRTN* was 0.335 for TTN. The magnitudes of the estimated additive effects for eight SNPs were between 0.10 and 0.14, 12 SNPs had additive effects that ranged from 0.05 to 0.10 and 12 SNPs had estimated effects that were <0.05 (Table 3).

**Table 3:** Results of additive effects of SNPs in the validation population (n = 5453) for TTN with positions based on *S. scrofa* build 10.2

Chr.	Position	SNP name	Additive effect	Previous association	Previous references
1	181,741,697	ASGA0005093	0.116	TTN^a^	
2	59,489,740	INRA0008845	−0.073	LTN, MAX	
2	81,675,738	ALGA0014021	−0.031	RTN	
3	49,321,597	H3GA0009450	0.059	MAX	[6, 11, 13]
3	134,660,431	MARC0090699	0.069	LTN	[11, 13]
4	25,899,175	DRGA0004616	−0.103	LTN	[11, 40]
4	33,780,262	ALGA0024379	0.031	RTN, TTN	[11, 40]
5	252,858	H3GA0017369	−0.061	RTN	[34]
6	157,649,704	M1GA0009139	−0.071	LTN	[11, 50]
7	103,208,408	*VRTN*/NV090	0.335	LTN, MAX, RTN, TTN	[6, 12, 13, 16, 17, 50]
7	124,146,658	MARC0073407	−0.039	LTN	[11]
8	16,445,414	ALGA0046611	0.077	TTN	[12, 51]
8	37,492,529	ALGA0047617	−0.127	LTN, MAX	[13]
10	51,681,377	DIAS0002581	−0.034	LTN	[13, 33–35, 43]
10	52,456,152	H3GA0030271	−0.062	LTN, MAX, RTN, TTN	[13, 33–35, 43]
10	52,679,135	MARC0018399	0.137	LTN, MAX, RTN, TTN	[13, 33–35, 43]
10	56,365,810	ASGA0103067	0.027	MAX	[11, 13, 33, 34]
10	58,071,987	ASGA0048302	0.029	MAX	[11, 13, 33, 34]
10	60,511,977	ASGA0048404	−0.044	MAX, RTN, TTN	[11, 33, 34]
12	26,420,000	ALGA0065784	−0.038	MAX	[11, 33, 34]
13	146,433,577	H3GA0037388	−0.123	LTN, MAX	
14	29,780,586	M1GA0018459	0.123	LTN, TTN	
14	41,043,761	ASGA0062848	0.106	LTN	
14	51,173,806	ASGA0063286	0.017	RTN	
14	52,942,907	ALGA0077532	0.016	RTN	
14	53,370,377	ASGA0063370	−0.062	MAX, RTN	
14	54,744,215	H3GA0040220	0.093	MAX, RTN, TTN	
14	54,791,585	ASGA0063388	NA^a^	MAX, RTN, TTN	
14	54,867,498	MARC0059175	NA^a^	MAX, RTN, TTN	
14	55,003,669	ASGA0063395	0.125	MAX, RTN	
14	55,429,701	ASGA0063406	NA	MAX, RTN	
15	37,205,130	ASGA0069274	−0.030	LTN	[50]
16	35,144,613	ALGA0090150	0.068	TTN	[11, 52]
16	50,977,092	MARC0028125	0.083	MAX	[11, 52]
18	4,400,270	ASGA0095800	−0.063	MAX	[13]
18	22,976,763	ALGA0097407	0.039	LTN	[53]

*LTN* left side teat number, *MAX* maximum teat number of a side, *RTN* right side teat number, *TTN* total teat number

^a^This SNP was not analyzed due to high linkage disequilibrium with another SNP included in the analysis

^b^Trait which originally exceeded the 1.4% genomic variation

## Discussion

How the development of the mammary gland has evolved is an interesting issue. It was observed by Aristotle more than 2000 years ago (as referenced by Diamond [27]) and shown more recently by Gilbert [28] that natural selection results in a number of teats equal to the maximum litter size expected, which is approximately twice the average litter size. However, selection for increased fecundity in livestock species (primarily swine and sheep) has resulted in litter sizes that exceed lactational capacity for many litters, thus requiring artificial rearing and/or cross-fostering of young to increase survival. Therefore, in these species, selective pressure needs to be placed on lactational capacity to increase neonatal survival and reduce the cost of production. Understanding the genetic mechanisms that regulate mammary gland development and teat number will contribute to the design of an optimal strategy for selection.

### Genetic factors that control teat number

We detected several interesting genomic regions that affect number of teats in commercial-type pigs. Foremost, was the confirmation of the association between SNPs in *VRTN* and number of teats. This association was also found by Ding et al. [6] and Duijvesteijn et al. [13]. While these two groups speculated that teat number and vertebra number are controlled by a similar set of genes, none of the regions reported in Table 3 for TTN were found to be associated with vertebra number in this population [23]. However, two regions for individual side counts did overlap with QTL for vertebra number, i.e. SSC5:0 and SSC12:26 Mb. The region on SSC5:0 Mb which explained 5.53% of the genomic variation for RTN is adjacent to the region that explained 2.22% of the genomic variation for thoracic vertebra number. Two candidate genes in this region include *ceramide kinase* (*CERK*) which produces ceramide-1-phosphate and has a role in cell proliferation and migration [29] and *CELSR1*, a regulator of planar cell polarity [30]. The region on SSC12:26 Mb explained 2.27% of the genomic variation for MAX, 8.59% of the genomic variation for lumbar vertebra number and 4.74% of the genomic variation for thoracolumbar vertebra number. This region is within the *COL1A1* gene. Mutations in *COL1A1* cause osteogenesis imperfecta leading to reduced bone mass and increased fracture. Two other potential candidate genes are the homeobox proteins *DLX3* and *DLX4* at 26.2 Mb on SSC12 but, to our knowledge, no role of these genes in mammary gland development has been described. *DLX3* induces the degradation of p63 [31] a transcription factor that is necessary for epidermal–mesenchymal interactions during embryonic development. Mice that lack p63 have no mammary glands [32]. In spite of the large contributions of these regions in the GWAS, their additive effects in the validation phase of this study were extremely low (0.061 and 0.038, respectively).

The region with the most consistent and largest effects on all teat count traits (SSC10:52) was also found by Duijvesteijn et al. [13] in Large White pigs as well as in several studies that used Meishan by occidental F_2_ populations [33–35]. This region contains the candidate genes *MPP7* and *FRMD4A*. *FRMD4A* resides within a copy number variation (CNV) region [36] and both *FRMD4A* and *MPP7* regulate the polarization of epithelial cells [37, 38]. The region on SSC10:60 Mb was reported by Guo et al. [11] who suggested *PLXDC2* as a possible candidate gene, which encodes a transmembrane receptor for the neurotrophic factor PEDF [39]. Hirooka et al. [33] and Rodríguez et al. [34] reported broad QTL intervals that spanned all of the 1-Mb windows on SSC10 reported in the current study.

The region on SSC4:25 Mb explained a high percentage of the genomic variation for LTN and had an additive effect on TTN of 0.103 in the validation population. Guo et al. [11] and Tortereau et al. [40] reported that this region segregated in Meishan cross populations. A potential candidate gene is *TRPS1*, which encodes a transcriptional repressor that regulates epithelial-mesenchymal transition [41] is required for morphogenesis during embryonic mammary gland development [42].

The region on SSC14:51–55 Mb has not been associated with teat number in pigs based on QTLdb and no obvious positional candidate genes were identified. This region had a large effect on RTN and MAX and the association with TTN was validated in the larger population. The SNP with the greatest estimated effect on TTN in the validation data was located at 55.00 Mb on SSC15 only 70 kb from the *T*-*box 1 transcription factor* gene (54.93 Mb). T-box transcription factors are critically important for normal tissue and organ development in the embryo. GWAS results for this region revealed a broad peak that spans several Mb. Several of the SNPs tested in the validation phase were in high linkage disequilibrium which made it difficult to directly pinpoint which SNP had the largest effect. Thus, multiple causative genes/variants are possible.

Other novel QTL regions with estimated additive effects exceeding 0.10 were on SSC1:181, SSC8:37, SSC13:146, SSC14:29 and SSC14:41 Mb. Unfortunately, identification of obvious candidate genes was unsuccessful. Although the SSC1:181 Mb region includes two genes for multiple epidermal growth factors (*MEGF6* and *MEGF11*) and the SSC14:41 Mb region contains two genes that are involved in the modulation of the NOTCH signaling pathway (*DTX1* and *RITA1*), these genes have not been shown to affect mammary gland development.

An unexpected finding is that among the eight regions discussed above, all with additive effects >0.1 in the validation population, five (SSC4:25, SCC8:37, SCC10:52, SCC13:146, and SCC14:55 Mb) were identified to have CNV segregating within the studied population [36]. Additional research is necessary to validate these CNV, determine their inheritance and test for association with teat counts.

### Mammary gland development in the pig

The concept that additional mammary glands arise from somite division in the developing embryo is supported by the effects of the *VRTN* gene that were observed on vertebra number and teat count [6, 12, 16, 17, 23] and of the *NR6A1* gene on SSC1 [6]. However, since genetic variation within *NR6A1* is only observed in crosses between Asian and European breeds, we were not able to evaluate this result. In spite of these two co-localizations of vertebra number and teat count in the proximity of *VRTN* and *NR6A1* as well as the speculation mentioned in Duijvesteijn et al. [13], none of the regions which exceeded 1% of the genomic variation for TTN (Fig. 1) were associated with vertebra number in this population [23]. The only region in the validation phase associated with vertebra number [23] was SSC12:26 Mb, which it had an extremely low additive effect (0.038). The region on SSC5:0 Mb was near a QTL for thoracic vertebra number (SSC5:1 Mb), which harbors *WNT7B* as candidate gene. However, there are clearly additional genetic factors that affect teat count since the USMARC Meishan population averages a 2.6 greater TTN [43] while having 1.5 fewer ribs and 2.0 fewer thoracolumbar vertebra than the Landrace–Duroc–Yorkshire population used in this GWAS.

Final teat number is likely a composite trait for which the underlying genetic model begins with somite division, followed either by proliferation of the mammary buds and/or regression of milk buds which results in teat number at birth. If genetic variation exists for all segments of the mammary gland development, then more progress may be possible if selection is applied to the component traits. This was the hypothesis on which was based the study of the maximum number of teats on one side since this would be the best estimate of an animal’s true genetic potential for the initial phase of somite proliferation. Measures of difference (L − R and DIF) may reflect regression of mammary buds. Based on the lack of genomic variation detected for L − R and DIF, these traits appear to be controlled by non-genetic factors. These factors must have a role during gestation, but the estimated effects of litter variation were zero for both L − R and DIF in the model development phase of this study. Similarly, Borchers et al. [8] and Fernández et al. [9] found virtually no effects of litter on L − R as well as only a minor effect of litter on count traits. While environmental factors such as stress and disease have been associated with fluctuating asymmetry in mammals [44], the factors that regulate asymmetrical mammary development in pigs is still unknown.

Few studies have actually evaluated left and right teat count values in pigs. While approximately 60% of pigs have the same number of teats on each side [8, 10] and the current study, a range of −3 to +3 for L − R was reported by Borchers et al. [8] and Fernández et al. [9]. A much wider range was observed in the current study (−4 to +7). Based on a limited number of studies, it appears that, in pig, teat number on the left side is a more heritable than teat number on the right side. Borchers et al. [8] reported a higher heritability for LTN than for RTN (0.20 vs. 0.18, respectively) although this difference was not statistically significant. Similarly, the current study found a 10% increase in heritability for LTN versus RTN in the model development dataset (Table 1) and in the subset of these animals used for GWAS (Table 2). Ding et al. [6] reported ten QTL for LTN and only seven QTL for RTN (30% fewer RTN than LTN) and 18 versus 13 (28% fewer RTN than LTN) 1-Mb regions explained more than 1% of the genomic variation in this study. The observation of identical trends and nearly identical magnitudes of differences is compelling. Furthermore, a region on SSC6:136 Mb that was reported by Ding et al. [6] to only affect LTN coincides with our results. In most studies [6, 9, 10] and the current study, the mean RTN was slightly larger than the mean LTN; however, this trend was not found by Borchers et al. [8]. Polythelia and polymastia were reported to occur more frequently on the right side in both humans [45] and mice [46] while missing mammary glands are most frequently observed on the left side in mice [46], thus the larger number of teats on the right side concurs with these phenomena. The current study indicates that MAX has an even stronger genetic component than either LTN or RTN. As this study is the first to report the genetic analysis of MAX, more studies are needed before this result can be confirmed because the differences in the estimated heritabilities were not statistically significant.

### Other factors that affect mammary gland development

Studies of embryonic development in mice have shown that mammary glands of male fetuses regress between day 13.5 and 15.5 of gestation due to circulating androgens, such that after birth male pups do not possess mammary gland tissue [47]. Based on this hypothesis, Drickamer et al. [48] studied the effect of the percentage of males in a litter on teat number in pigs and found that the mean teat number decreased as the percentage of male pigs increased. Some studies in pigs have included a fixed effect for sex of the pig when analyzing teat number but either they did not indicate if the effect was significant or they did not present estimates of the effect [8, 13]. Willham and Whatley [10] reported similar number of teats for male versus female piglets. Our data did not show any impact of the sex of the piglet (mean TTN of 14.73 for both male and female piglets) or of the percentage of males in a litter (regression coefficients were <0.001 for all analyses). In fact, a query on three populations of commercial-type pigs born at USMARC (representing sampling industry animals in the 1993, 2000 and 2010–2015) resulted in virtually identical mean numbers of teats among male and female piglets (14.64 vs. 14.65; respectively) among more than 110,000 piglets.

**Fig. 2:**
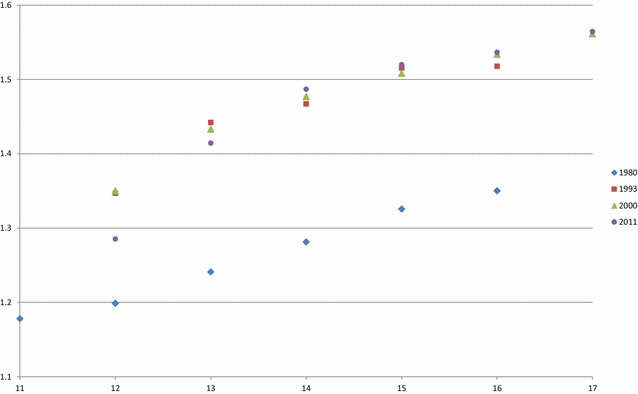
Average birth weight (kg) for piglets born by total number of teats from four different populations at USMARC derived from commercial genetics in 1980, 1993, 2000 and 2011. Each class has at least 800 animals represented in the mean value. No values for the 11 teat number class for 1993, 2000 or 2011 and the 17 teat number class for 1980 or 1993 are provided due to too few pigs

Borchers et al. [8] reported evidence that teat count was correlated with birth weight. In the USMARC populations studied here, this trend was also present (Fig. 2) in all three commercial-type populations that are maintained since the early 1990s as well as in a population created in 1980. Surprisingly, TTN was not associated with number of piglets born or vertebra numbers. Since mammary gland and teat development is already evident by 28 days of gestation, the causative factor(s) for the correlation between birth weight and TTN must occur in early stages of development. Based on the estimated litter variance for all analyzed traits, the factor(s) are not common to all fetuses within a litter. An improved understanding of these factors may enable increasing both teat number and birth weights in commercial swine.

### Selection for increased number of teats

Estimated heritabilities indicate that selection would be successful for increased teat number. This was documented in sheep by Alexander Graham Bell [49] who observed that some sheep were born with up to eight teats versus the normal 2. Selection pressure for teat number in swine has typically relied on a minimum threshold (independent culling level) where pigs that were above this threshold were selection candidates. Thresholds of either 12 or 14 were often implemented. A more dramatic increase in teat number could be obtained if more rigorous selection was applied.

Estimated heritabilities for teat number often range from 0.2 to 0.4 [8]. The estimated heritability presented in Table 1 (0.49) was higher than the genomic heritability from GWAS (0.31) and the estimated heritability in the validation phase when no SNPs were fit (0.37). A contributing factor to these differences was the animals included in the analyses. During the development of the model, all animals born were included. However, for all other analyses, only genotyped animals were included. Most of the genotyped animals were females that had been retained for breeding so a minimum of 12 teats were required. As shown by the differences between minimum and maximum values in Tables 1 and 2, less phenotypic variation was present in the genotyped animals and the genetic variation estimated in these animals was lower. Arakawa [50] found a lower heritability based on SNPs (0.34) than that estimated from pedigree information (0.43), which is similar to the current study.

If final teat number is the result of the number of mammary buds initially developed and a maintenance (or regression) component, which acts randomly relative to the side of the developing organism, then MAX would be a better indicator of the initial number of mammary buds than RTN or LTN. L − R or DIF could predict the maintenance or regression component; however, genetic variation for these traits was nil, thus selection based on these measures would likely be ineffective. Among the traits analyzed here, TTN is the trait with the highest heritability and the most phenotypic variation and since it is the most important trait for swine production, selective pressure to increase lactational capacity in commercial sows should focus on TTN. Based on the association between birth weight and teat number, this would result in a serendipitous increase of piglet birth weight if this association is due to genetics.

## Conclusions

Selection to increase the number of teats is possible in pigs and use of genetic markers should expedite progress. Since individual side counts of teats is not commonly recorded, exploitation of loci that independently control RTN or LTN would be most effective with genetic markers. These results validate the effect of *VRTN* on teat number as well as the QTL located on SSC4:25 and SSC10:52 Mb, and we identified an important novel region on SSC14:51–55 Mb, which needs to be further studied. The most heritable trait that also possessed the most phenotypic variation was TTN. Therefore, without SNPs, single-trait traditional selection for TTN would yield the greatest gains; however, a selection index including TTN and MAX might slightly improve selection response. In this study, factors such as sex of the pig or of other pigs in the litter had no effect on TTN and there was no common environmental effect associated with fluctuating asymmetry for teat counts. The observed correlation between birth weight and teat number was unexpected and deserves further investigation. While it is clear that teat number can be increased in pigs, whether an increase in number of teats will result in increased total milk production still needs to be addressed.

## Additional file

**Additional file 1: Table S1**. Information on 1-Mb windows that explained more than 0.4% of the genomic variation as determined by GENSEL. Description: A listing of the information from all 1-Mb windows (defined in SSC and Mb) that exceeded 0.4% of the genomic variation as determined by GENSEL. The number of SNPs within each window (#SNPs), percentage of genomic variation explained by the SNPs on average across all post-burn-in samples (%Var), the frequency at which a 1-Mb window explained more than the average amount of genomic variation (p > Average), the position of the first SNP (map_pos0) and last SNP (map_posn) in the window. The last eight columns pertain to the SNPs within the 1-Mb window with the largest estimated effect: SNP name, location in build 10.2, effect size, standard error of the estimate, frequency of SNP retention in each sample, allele frequency of the B allele in the population, T-test for the effect and simple P-value corresponding to the T-test.

## References

[1] Andersen IL, Nævdal E, Bøe KE (2011). Maternal investment, sibling competition, and offspring survival with increasing litter size and parity in pigs (*Sus scrofa*). Behav Ecol Sociobiol.

[2] Merks JWM, Mathur PK, Knol EF (2012). New phenotypes for new breeding goals in pigs. Animal.

[3] Nielsen B, Su G, Lund MS, Madsen P (2013). Selection for increased number of piglets at d 5 after farrowing has increased litter size and reduced piglet mortality. J Anim Sci.

[4] Cassady JP, Young LD, Leymaster KA (2002). Heterosis and recombination effects on pig reproductive traits. J Anim Sci.

[5] Kim JS, Jin DI, Lee JH, Son DS, Lee SH, Yi YJ (2005). Effects of teat number on litter size in gilts. Anim Reprod Sci.

[6] Ding N, Guo Y, Knorr C, Ma J, Mao H, Lan L (2009). Genome-wide QTL mapping for three traits related to teat number in a White Duroc × Erhualian pig resource population. BMC Genet.

[7] Pumfrey RA, Johnson RK, Cunningham PJ, Zimmerman DR (1980). Inheritance of teat number and its relationship to maternal traits in swine. J Anim Sci.

[8] Borchers N, Reinsch N, Kalm E (2002). Teat number, hairiness and set of ears in a Piétrain cross: variation and effects on performance traits. Arch Tierz Dummerstorf.

[9] Fernández A, Toro M, Rodríguez C, Silió L (2004). Heterosis and epistasis for teat number and fluctuating asymmetry in crosses between Jiaxing and Iberian pigs. Heredity.

[10] Willham RL, Whatley JA (1962). Genetic variation in nipple number in swine. J Anim Breed Genet.

[11] Guo YM, Lee GJ, Archibald AL, Haley CS (2008). Quantitative trait loci for production traits in pigs: a combined analysis of two Meishan × Large White populations. Anim Genet.

[12] Sato S, Atsuji K, Saito N, Okitsu M, Sato S, Komatsuda A (2006). Identification of quantitative trait loci affecting corpora lutea and number of teats in a Meishan × Duroc F2 resource population. J Anim Sci.

[13] Duijvesteijn N, Veltmaat JM, Knol EF, Harlizius B (2014). High-resolution association mapping of number of teats in pigs reveals regions controlling vertebral development. BMC Genomics.

[14] Mikawa S, Morozumi T, Shimanuki S-I, Hayashi T, Uenishi H, Domukai M (2007). Fine mapping of a swine quantitative trait locus for number of vertebrae and analysis of an orphan nuclear receptor, germ cell nuclear factor (NR6A1). Genome Res.

[15] Mikawa S, Sato S, Nii M, Morozumi T, Yoshioka G, Imaeda N (2011). Identification of a second gene associated with variation in vertebral number in domestic pigs. BMC Genet.

[16] Lopes MS, Bastiaansen JWM, Harlizius B, Knol EF, Bovenhuis H (2014). A genome-wide association study reveals dominance effects on number of teats in pigs. PLoS One.

[17] Verardo LL, Silva FF, Lopes MS, Madsen O, Bastiaansen JWM, Knol EF (2016). Revealing new candidate genes for reproductive traits in pigs: combining Bayesian GWAS and functional pathways. Genet Sel Evol.

[18] Veltmaat JM, Ramsdell AF, Sterneck E (2013). Positional variations in mammary gland development and cancer. J Mammary Gland Biol Neoplasia.

[19] Propper AY, Howard BA, Veltmaat JM (2013). Prenatal morphogenesis of mammary glands in mouse and rabbit. J Mammary Gland Biol Neoplasia.

[20] Veltmaat JM, Relaix F, Le LT, Kratochwil K, Sala FG, van Veelen W (2006). Gli3-mediated somitic Fgf10 expression gradients are required for the induction and patterning of mammary epithelium along the embryonic axes. Development.

[21] Schneider JF, Rempel LA, Rohrer GA (2012). Genome-wide association study of swine farrowing traits. Part I: genetic and genomic parameter estimates. J Anim Sci.

[22] Meyer K (2007). WOMBAT: a tool for mixed model analyses in quantitative genetics by restricted maximum likelihood (REML). J Zhejiang Univ Sci B.

[23] Rohrer GA, Nonneman DJ, Wiedmann RT, Schneider JF (2015). A study of vertebra number in pigs confirms the association of vertnin and reveals additional QTL. BMC Genet.

[24] Groenen MAM, Archibald AL, Uenishi H, Tuggle CK, Takeuchi Y, Rothschild MF (2012). Analyses of pig genomes provide insight into porcine demography and evolution. Nature.

[25] Sargolzaei M, Chesnais JP, Schenkel FS (2014). A new approach for efficient genotype imputation using information from relatives. BMC Genomics.

[26] Ramos AM, Crooijmans RPMA, Affara NA, Amaral AJ, Archibald AL, Beever JE (2009). Design of a high density SNP genotyping assay in the pig using SNPs identified and characterized by next generation sequencing technology. PLoS One.

[27] Diamond JM (1987). Evolutionary adaptations. Aristotle’s theory of mammalian teat number is confirmed. Nature.

[28] Gilbert AN (1986). Mammary number and litter size in Rodentia: the “one-half rule”. Proc Natl Acad Sci USA.

[29] Hoeferlin LA, Wijesinghe DS, Chalfant CE (2013). The role of ceramide-1-phosphate in biological functions. Handb Exp Pharmacol.

[30] Tissir F, Goffinet AM (2013). Atypical cadherins Celsr1-3 and planar cell polarity in vertebrates. Prog Mol Biol Transl Sci.

[31] Di Costanzo A, Festa L, Duverger O, Vivo M, Guerrini L, La Mantia G (2009). Homeodomain protein Dlx3 induces phosphorylation-dependent p63 degradation. Cell Cycle.

[32] Mills AA, Zheng B, Wang X-J, Vogel H, Roop DR, Bradley A (1999). *p63* is a *p53* homologue required for limb and epidermal morphogenesis. Nature.

[33] Hirooka H, de Koning DJ, Harlizius B, van Arendonk JAM, Rattink AP, Groenen MAM (2001). A whole-genome scan for quantitative trait loci affecting teat number in pigs. J Anim Sci.

[34] Rodríguez C, Tomás A, Alves E, Ramirez O, Arqué M, Muñoz G (2005). QTL mapping for teat number in an Iberian-by-Meishan pig intercross. Anim Genet.

[35] Dragos-Wendrich M, Moser G, Bartenschlager H, Reiner G, Geldermann H (2003). Linkage and QTL mapping for *Sus scrofa* chromosome 10. J Anim Breed Genet.

[36] Wiedmann RT, Nonneman DJ, Rohrer GA (2015). Genome-wide copy number variations using SNP genotyping in a mixed breed swine population. PLoS One.

[37] Ikenouchi J, Umeda M (2010). FRMD4A regulates epithelial polarity by connecting Arf6 activation with the PAR complex. Proc Natl Acad Sci USA.

[38] Stucke VM, Timmerman E, Vandekerckhove J, Gevaert K, Hall A (2007). The MAGUK protein MPP7 binds to the polarity protein hDlg1 and facilitates epithelial tight junction formation. Mol Biol Cell.

[39] Cheng G, Zhong M, Kawaguchi R, Kassai M, Al-Ubaidi M, Deng J (2014). Identification of PLXDC1 and PLXDC2 as the transmembrane receptors for the multifunctional factor PEDF. Elife.

[40] Tortereau F, Gilbert H, Heuven HCM, Bidanel JP, Groenen MAM, Riquet J (2010). Combining two Meishan F2 crosses improves the detection of QTL on pig chromosomes 2, 4 and 6. Genet Sel Evol.

[41] Huang JZ, Chen M, Zeng M, Xu SH, Zou FY, Chen D (2016). Down-regulation of TRPS1 stimulates epithelial–mesenchymal transition and metastasis through repression of *FOXA1*. J Pathol.

[42] Boras-Granic K, Chang H, Grosschedl R, Hamel PA (2006). Lef1 is required for the transition of Wnt signaling from mesenchymal to epithelial cells in the mouse embryonic mammary gland. Dev Biol.

[43] Rohrer GA (2000). Identification of quantitative trait loci affecting birth characters and accumulation of backfat and weight in a Meishan–White composite resource population. J Anim Sci.

[44] Møller AP (2006). A review of developmental instability, parasitism and disease: infection, genetics and evolution. Infect Genet Evol.

[45] Schmidt H (1998). Supernumerary nipples: prevalence, size, sex and side predilection—a prospective clinical study. Eur J Pediatr.

[46] Little CC, McDonald H (1945). Abnormalities of the mammae in the house mouse. J Hered.

[47] Veltmaat JM, Mailleux AA, Thiery JP, Bellusci S (2003). Mouse embryonic mammogenesis as a model for the molecular regulation of pattern formation. Differentiation.

[48] Drickamer LC, Rosenthal TL, Arthur RD (1999). Factors affecting the number of teats in pigs. J Reprod Fertil.

[49] Castle WE (1924). The genetics of multi-nippled sheep: an analysis of the sheep-breeding experiments of Dr. and Mrs. Alexander Graham Bell at Beinn Bhreagh, N. S. J Hered.

[50] Arakawa A, Okumura N, Taniguchi M, Hayashi T, Hirose K, Fukawa K (2015). Genome-wide association QTL mapping for teat number in a purebred population of Duroc pigs. Anim Genet.

[51] Cassady JP, Johnson RK, Pomp D, Rohrer GA, Van Vleck LD, Spiegel EK (2001). Identification of quantitative trait loci affecting reproduction in pigs. J Anim Sci.

[52] Bidanel JP, Rosendo A, Iannuccelli N, Riquet J, Gilbert H, Caritez JC (2008). Detection of quantitative trait loci for teat number and female reproductive traits in Meishan × Large White F2 pigs. Animal.

[53] Hernandez SC, Finlayson HA, Ashworth CJ, Haley CS, Archibald AL (2014). A genome-wide linkage analysis for reproductive traits in F2 Large White × Meishan cross gilts. Anim Genet.

